# En-bloc resection of thoracic calcified meningioma with inner dural layer in recapping T-saw laminoplasty: a case report

**DOI:** 10.1186/s12893-015-0066-5

**Published:** 2015-07-04

**Authors:** Naohisa Miyakoshi, Michio Hongo, Yuji Kasukawa, Yoichi Shimada

**Affiliations:** Department of Orthopedic Surgery, Akita University Graduate School of Medicine, 1-1-1 Hondo, Akita, 010-8543 Japan

**Keywords:** Bone union, Dural layer, Recapping laminoplasty, Spinal meningioma, T-saw

## Abstract

**Background:**

In the treatment of spinal cord tumors, complete tumor resection with complete reconstruction of the spine represents the ideal goal. However, for the treatment of heavily calcified spinal meningioma, en-bloc resection of the tumor together with the involved dura at the tumor base through laminectomy is quite difficult. Conventional laminectomy is not wide enough to allow full exposure of such tumors, and postoperative dural defects can cause serious complications.

**Case presentation:**

A 58-year-old Japanese woman presented with a 3-month history of progressive muscle weakness and numbness of the lower extremities. Magnetic resonance imaging (MRI) and computed tomography showed a calcified spinal cord tumor with dural tail sign at the T10-T11 level, severely compressing the spinal cord anterolaterally. The patient underwent en-bloc resection of the tumor with the inner layer of the dura and preservation of the outer layer of the dura through recapping T-saw laminoplasty of T10-T11, including bilateral facet joints. The tumor was histologically diagnosed as meningioma. Postoperatively, the patient achieved complete recovery of neurological functions. Bone union after laminoplasty was obtained within 6 months. Follow-up MRI at 5 years postoperatively demonstrated no recurrence of the tumor.

**Conclusion:**

Resection of spinal meningioma with only the inner layer of dura can minimize postoperative complications, including spinal fluid leakage. Recapping T-saw laminoplasty provides extensive exposure for tumor removal. The combination of these techniques may offer an ideal surgical option for complete resection of spinal meningioma with complete preservation of the posterior spinal elements.

## Background

With conventional methods for surgical resection of spinal meningiomas, either the involved dura at the tumor base is completely resected together with the tumor, or extensive coagulation alone is applied to the tumor base of the dura after tumor removal [1]. Saito et al. [1] reported a third technique involving resection of the meningioma with the inner layer of the dura representing the base of the tumor. This technique is simple, is expected to reduce the risk of recurrence compared to coagulation alone, and does not require any artificial dura grafting, since the outer layer of dura is preserved for dural closure.

For thoracic spinal cord tumor resection, laminectomy is usually introduced. However, potential problems include invasion of hematoma and scar tissue into the spinal canal, postoperative instability, and subluxation or kyphotic deformity of the spine [2, 3]. In addition, conventional laminectomy does not usually allow good intraoperative visualization, and en-bloc resection of spinal meningioma is sometimes difficult, particularly for large or hard tumors with extensive calcification.

Recapping T-saw laminoplasty cutting from the pedicle to the transverse process can provide extremely wide exposure of the spinal canal, and is indicated for spinal cord tumors in the thoracic or lumbar spine [4]. Using this technique, we can observe the lateral surface of the dura, and the excised posterior spinal elements can be replaced in perfect in-situ apposition in the anatomical position. The threadwire saw (T-saw) was developed by Tomita and Kawahara as a thin, smooth, and flexible bone-cutting device [5]. As bone loss from use of the T-saw cut is negligible, the excised laminae can be restored to the exact original anatomical position [4].

Kawahara et al. [4, 6] reported successful results from recapping T-saw laminoplasty for spinal cord tumors, but those cases did not include any examples of meningioma resected with the inner dural layer and preservation of the outer dural layer. We report herein our clinical experience with treating a calcified spinal meningioma in the thoracic spine that was successfully excised with only the inner layer of the dura through recapping T-saw laminoplasty. The combination of these two techniques for the treatment of spinal meningioma has not previously been described in the English literature.

## Case presentation

A 58-year-old Japanese woman presented with a 3-month history of progressive walking disturbance due to muscle weakness and numbness in both legs. She also complained of urinary incontinence. Neurological examination showed left-side-dominant weakness (i.e., power as evaluated by manual muscle testing: 2-3/5 on left leg, 3-4/5 on right leg), hypesthesia 3 cm below the umbilicus, loss of deep sensation below both knees, and hyperreflexia of both lower extremities.

**Fig. 1:**
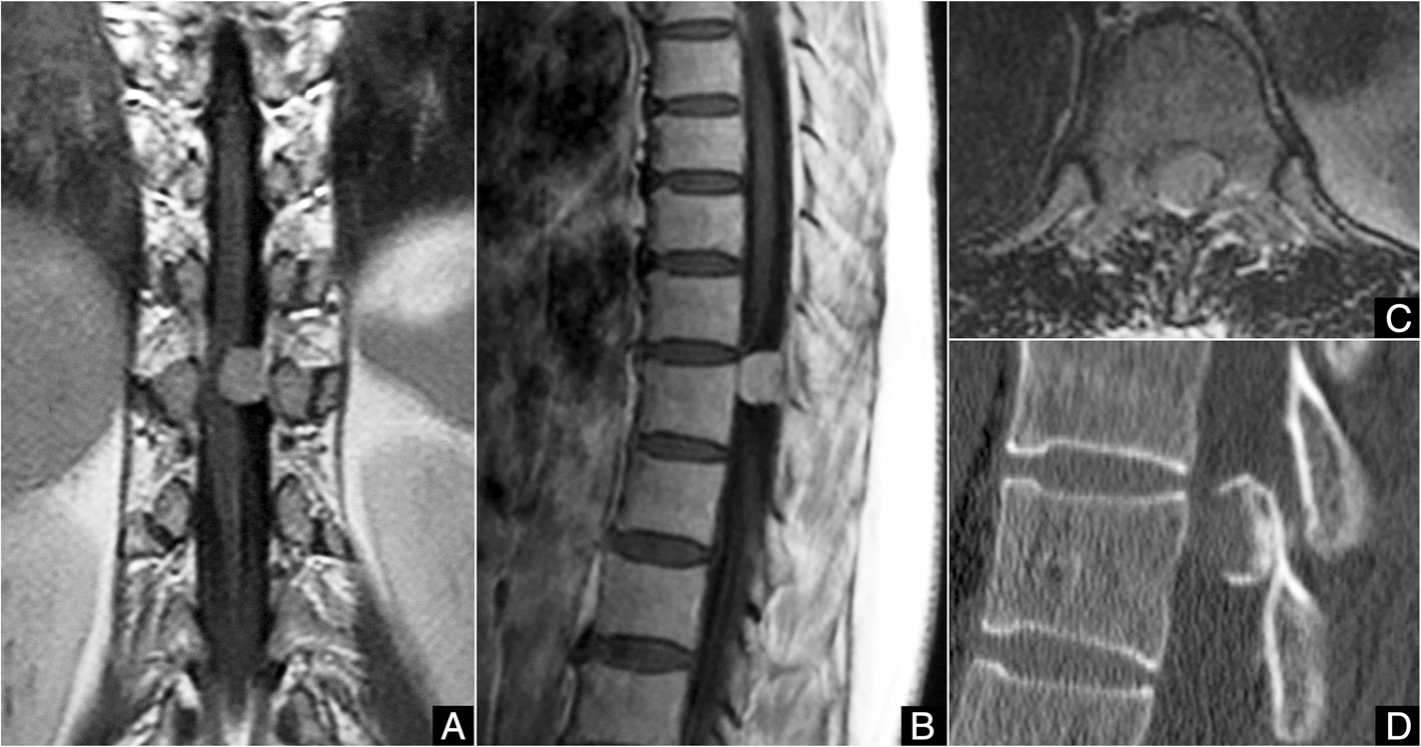
Preoperative gadolinium-enhanced T1-weighted magnetic resonance imaging (MRI) and computed tomography (CT) of the thoracic spine. **a** MRI in the coronal orientation, **b** MRI in the sagittal orientation, and **c** MRI in the axial orientation showing an intradural extramedullary tumor with dural tail sign located on the left and dorsal sides at the T10-T11 level, compressing the spinal cord severely. **d** Sagittal reconstruction CT shows extensive peripheral calcification of the tumor

Magnetic resonance imaging (MRI) of the thoracic spine demonstrated an intradural extramedullary tumor located posterolaterally on the left side at the T10-T11 level, compressing the spinal cord severely to the ventral and right side. Contrast-enhanced MRI showed marked homogeneous enhancement with a dural tail sign (Fig. 1a-c). Computed tomography (CT) of the spine showed heavy peripheral calcification of the tumor (Fig. 1d). Based on these imaging findings, hard spinal meningioma with calcification located posterolateral to the spinal cord was diagnosed. For safe, complete, en-bloc resection of the tumor under good exposure, to minimize the risk of postoperative complications due to dural defect, and for complete preservation of the posterior spinal elements, we planned to resect this tumor with only the inner dural layer while preserving the outer dural layer through recapping T-saw laminoplasty.

**Fig. 2:**
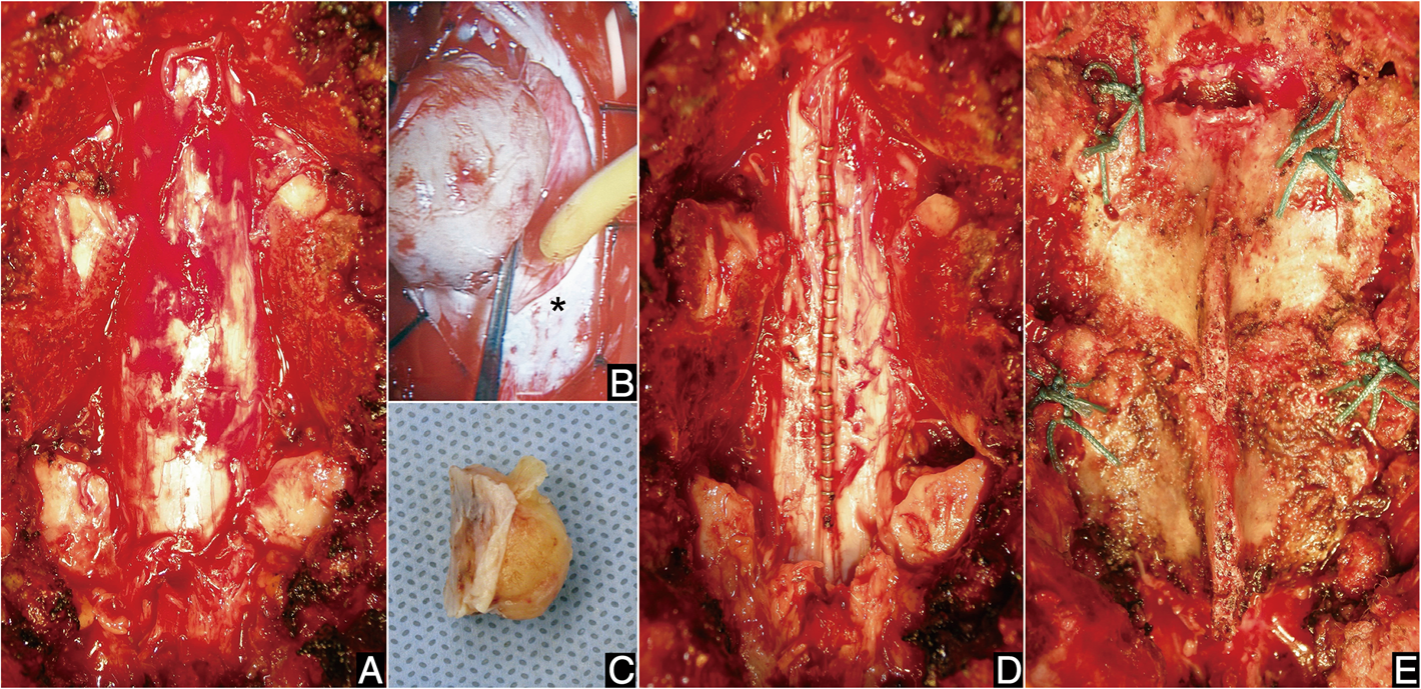
Intraoperative photographs. **a** Dura was widely exposed after en-bloc excision of the posterior elements of T10-T11, including bilateral T10-T11 facet joints, by cutting the pars interarticularis of the lamina of T10 and pedicles to the transverse processes of T11 using a T-saw. **b** After opening the outer layer of dura, the inner layer of dura (asterisk) surrounding the tumor was cut and the tumor was lifted up. **c** The tumor was resected en bloc with the inner layer of dura. **d** The outer layer of dura was closed with titanium clips. **e** Following complete resection of the tumor and repair of the outer layer of dura, the excised posterior elements were recapped exactly to their anatomical sites and sutured

Surgery was performed under transcranial electrical stimulation of motor evoked potentials for spinal cord monitoring. The spinous process of the above vertebra (T9) was split longitudinally and divided at its base from the lamina with bilateral paravertebral muscles attached to the split spinous process. The T10-T11 posterior arches lateral to bilateral transverse processes were then exposed. Bilateral pars interarticularis of the lamina of T10 and bilateral pedicles to the transverse processes of T11 were cut using a T-saw (Koshiya, Kanazawa, Japan). Posterior elements of T10-T11, including bilateral T10-T11 facet joints, were excised en bloc and the posterior and lateral surfaces of the dura were widely exposed (Fig. 2a). After exposing the dura, an incision was made only within the outer layer of the dura under microscopy, and the outer layer was then stripped away from the inner layer [1]. After retracting the outer layers bilaterally, an incision was made in the inner layer and continued around the tumor base [1]. The hard calcified tumor was then resected en bloc with the inner layer of dura (Fig. 2b, c). The outer layer of dura was closed with titanium clips (Fig. 2d). The excised T10-T11 laminae were then recapped exactly to the anatomical sites and the corresponding cut surfaces were bound by sutures (Fig. 2e). The split T9 spinous process was reapproximated and the paravertebral muscles were closed. Histopathological study of the tumor was consistent with calcified meningioma. This surgery was conducted in accordance with the Declaration of Helsinki.

**Fig. 3:**
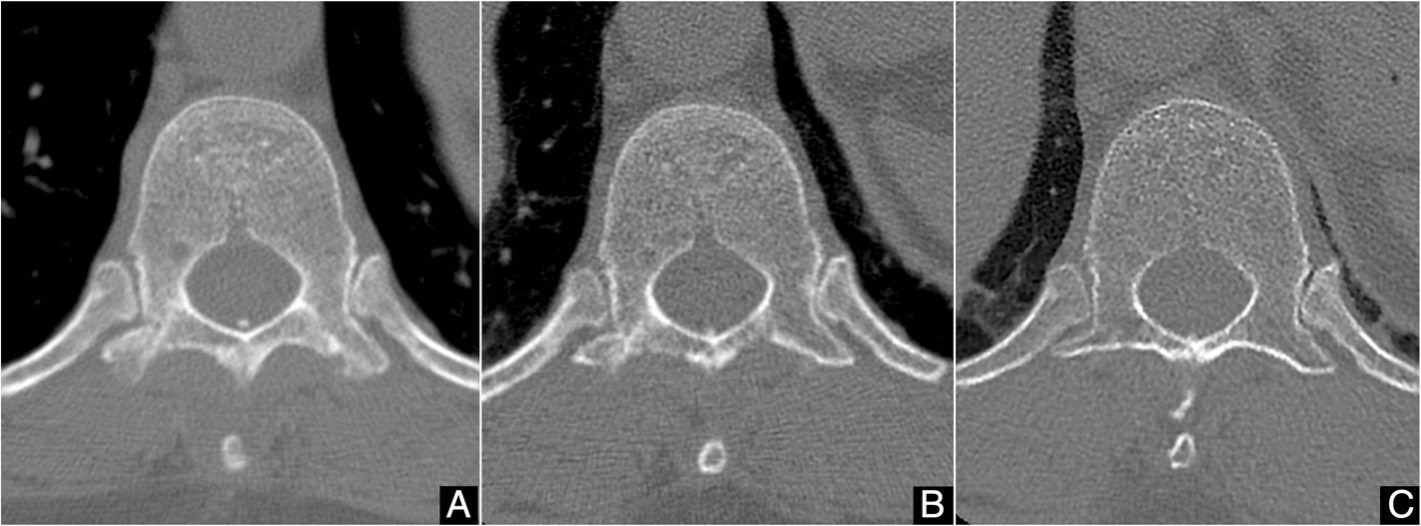
Postoperative computed tomography at the T11 level. **a** Cut lines between the recapped lamina and host bone at 1 month after surgery. **b** Bony union is apparent at 6 months after surgery. **c** Intact spinal canal is confirmed at 5 years after surgery

**Fig. 4:**
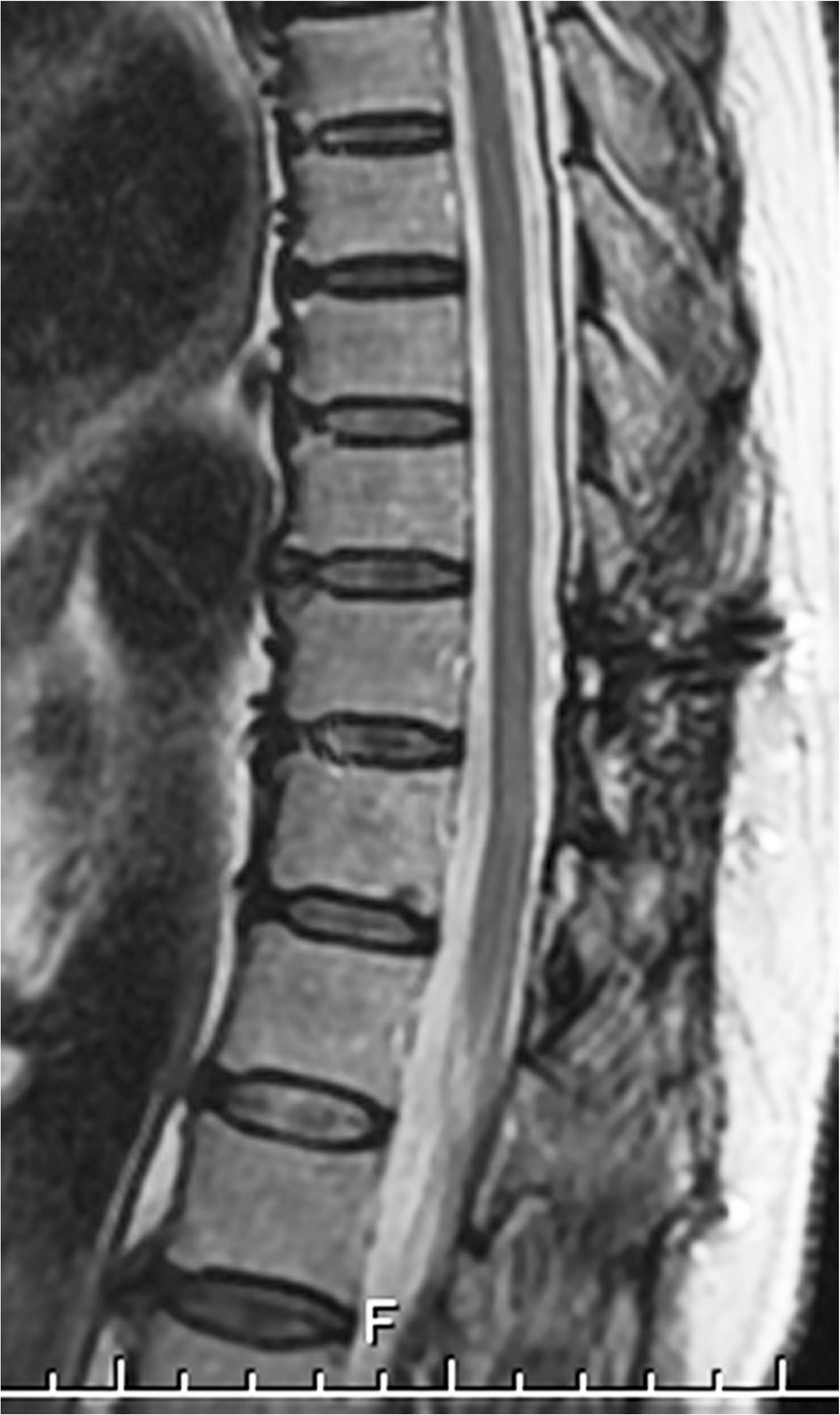
Postoperative sagittal T2-weighted imaging obtained 5 years after surgery. No recurrence of the tumor is evident

The postoperative course was uneventful and the patient gradually recovered all neurological functions. Motor and sensory functions of the lower extremities and bladder function showed complete normalization within 2 weeks. The patient wore a thoraco-lumbo-sacral orthosis for 3 months. CT confirmed primary bone union within 6 months (Fig. 3). Postoperative instability has not occurred as of the latest follow-up at 5 years after surgery. MRI 5 years postoperatively showed no recurrence of the tumor (Fig. 4).

## Discussion

Saito et al. [1] reported three cases of spinal meningioma successfully resected with the inner layer of dura and preservation of the outer layer. With this technique, they reported no recurrences within a follow-up duration ranging from 4 months to 2 years and 10 months [1]. The risk of tumor recurrence with this technique remains unknown, because long-term follow-up of a large series has not yet been reported. However, Saito et al. [1] histologically proved that the preserved outer layer of dura did not demonstrate the presence of any tumor cells. Thus, if the outer layer of dura is easily stripped, the risk of tumor infiltration into this layer may be minimal. Although longer follow-up is needed, the present case showed no recurrence after 5 years of follow-up. However, leaving the outer layer of dura that is in contact with the lesion may not be prudent in some cases. The need for dural resection or preservation must be balanced against the risk of recurrence. Intraoperative, histological confirmation of the absence of tumor cells using frozen sections represents a potentially useful option for future cases.

Previous studies have shown that calcified thoracic meningiomas were more likely to adhere to the nerves and dura, a finding that might explain the high incidence of neurological dysfunction and spinal fluid leakage after surgery [7]. Saito et al. [1] mentioned that resection of meningioma with the inner dural layer cannot be applied if the tumor is located anterior to the spinal cord, or is heavily calcified. However, our experience suggests that heavy calcification as in the present case is not a contraindication for this procedure, as long as the outer dural layer can still be easily stripped.

Conventional laminectomy provides sufficient operative exposure for most spinal lesions, but several difficulties remain, including postoperative spinal deformity and symptomatic epidural scar formation. For example, the incidence of spinal deformity after laminectomy is reportedly 33-100 % [2]. To reduce these risks, laminoplasty has been recommended in several studies [3, 4, 8]. Laminoplasty is said to offer the requisite exposure and decompression of the spinal canal, while at the same time maintaining spinal stability and integrity of the posterior elements [2]. Several kinds of laminoplasties have been reported for lesions of the thoracic and lumbar spine, including use of a titanium miniplate and translaminar screws [3, 4, 9]. However, because exposure widths of the spinal canal with these laminoplasties resemble those of conventional laminectomy, we cannot explore more lateral sites up to the neural foramina using these methods. In cases with hard calcified meningioma located laterally, safe and good exposure to a more lateral extent is essential, but is impossible through conventional laminectomy or these laminoplasties. In this regard, recapping T-saw laminoplasty cutting from pedicle to transverse process provides excellent exposure and allows sufficient space for total excision of the tumor.

For recapping laminoplasty, use of a T-saw is important because bone loss from use of the T-saw cut is negligible, and the excised laminae can be restored to the exact original anatomical position [4]. This phenomenon contributes to high rates of bone union. A previous study of 24 patients who underwent spinal cord tumor resection through recapping T-saw laminoplasty showed a bone union rate of 96 % at 6 months after surgery and 100 % by 12 months after surgery [4]. The present case likewise revealed primary bone union within 6 months. Postoperative instability has not been encountered as of the latest follow-up, 5 years after surgery. However, further studies with long-term follow-up are needed to confirm that the risk of delayed instability is low.

Another merit of recapping T-saw laminoplasty is that the epidural scar tissue that sometimes results in deterioration of symptoms after surgery seems to be minimized. The development of epidural scar tissue (postlaminectomy membrane) is considered to result from several mechanisms, including invasion of the spinal canal by the erector spinae muscles [10]. Laminoplasties can thus be introduced to prevent the formation of postlaminectomy membrane. However, whether laminoplasty is better than laminectomy in preventing postlaminectomy scar formation remains controversial [11, 12]. Kuraishi et al. [11] reported a case with symptomatic epidural scar formation after cervical laminoplasty with hydroxyapatite spacer. Based on histopathological examination of the resected tissue, they concluded that the cause of this excessive epidural scar formation was foreign body reaction to the hydroxyapatite [11]. We think recapping T-saw laminoplasty can greatly minimize epidural scar formation compared to other techniques, because this technique can prevent muscular invasion into the spinal canal and does not use artificial materials such as hydroxyapatite or metals.

One possible complication of recapping T-saw laminoplasty is neural injury when the T-saw passes beneath the lamina [3]. This complication may occur in cases with solid, hard spinal cord tumors as in the present case. We thus suggest that recapping T-saw laminoplasty should be performed under intraoperative spinal cord monitoring, which has been shown to reduce neurological deterioration [13].

Recapping T-saw laminoplasty can be applied for any type of spinal cord tumor in the thoracic or lumbar spine. Dumbbell-type tumors or tumors with expansion into multiple foramina can also be resected using this technique [4, 14]. However, spinal tumors with severe epidural adhesion increase the risk of spinal cord injury when the T-saw passes beneath the lamina, and such cases should thus be considered contraindicated.

## Conclusions

Removal of spinal meningioma with the inner layer of dura and preservation of the outer layer of dura, through recapping T-saw laminoplasty, allows wide exposure, complete en-bloc resection of the tumor, primary dural repair without any artificial dura grafting, and complete preservation of posterior spinal elements after surgery. This offers a feasible combination technique for the treatment of spinal meningioma, even if the tumor is hardened by heavy calcification.

## Consent

Written informed consent was obtained from the patient for publication of this case report and the accompanying images. A copy of the written consent is available for review by the Editor of this journal.

## Abbreviations

CT: Computed tomography
MRI: Magnetic resonance imaging
T-saw: Threadwire saw
